# Molecular epidemiological investigation and recombination analysis of Cachavirus prevalent in China

**DOI:** 10.3389/fvets.2024.1375948

**Published:** 2024-05-01

**Authors:** Chaoliang Leng, Xiang Tian, Hongyue Zhai, Jun Ji, Lunguang Yao

**Affiliations:** Henan Provincial Engineering Laboratory of Insects Bio-reactor, Henan Provincial Engineering and Technology Center of Health Products for Livestock and Poultry, Henan Provincial Engineering and Technology Center of Animal Disease Diagnosis and Integrated Control, Nanyang Normal University, Nanyang, China

**Keywords:** Cachavirus, molecular epidemiological investigation, recombination analysis, *Chaphamaparvovirus*, diarrhea

## Abstract

*Chaphamaparvovirus carnivoran1* (canine *Chaphamaparvovirus*, also known as Cachavirus [CachaV]) is a novel parvovirus first reported in dog feces collected from the United States in 2017 and China in 2019. To continuously track its infection and evolution status, 276 canine anal swabs were obtained from pet hospitals in central, northern, and eastern China between 2021 and 2023 and screened via polymerase chain reaction; subsequently, a systematic study was conducted. Of these samples, nine (3.3%) were positive for CachaV. Using polymerase chain reaction, whole genome sequences of the nine CachaV-positive strains were amplified. The NS1 amino acid sequence identity between CachaV strains from China and other countries was 96.23–99.85%, whereas the VP1 protein sequence identity was 95.83–100%. CHN230521 demonstrated the highest identity for NS1 amino acids (99.85%) and VP1 amino acids (100%) with NWT-W88 and CP-T015. According to the model prediction of CHN220916-VP1 protein, Met64Thr, Thr107Ala, and Phe131Ser mutations may cause tertiary structural changes in VP1 protein. Interestingly, each of the nine CachaV strains harbored the same site mutations in NS1 (Ser252Cys, Gly253Leu, and Gly254Thr). Although no explicit recombination events were predicted, the clustering and branching of the phylogenetic tree were complicated. Based on the evolution trees for VP1 and NS1, the nine CachaV strains identified from 2021 to 2023 were closely related to those identified in gray wolves and cats. This study may be beneficial for evaluating the prevalence of CachaVs in China, thereby understanding the evolution trend of CachaVs.

## 1 Introduction

Parvoviruses are nonenveloped single-stranded DNA viruses with a small genome (4–5 kbp) (1, 2). Historically, parvoviruses infecting both vertebrate and invertebrate hosts were categorized into two subfamilies, namely, *Densovirinae* and *Parvovirinae*, respectively, which were further divided into eight genera (3). Recent research has identified new parvovirus variations in animals, directly leading to the reclassification of *Parvoviridae* (3–5). Currently, the International Committee on Taxonomy of Viruses has included a third subfamily into the family *Parvoviridae*, comprising *Parvovirinae*, *Densovirinae*, and *Hamaparvovirinae*, which further comprises five novel genera—*Penstylhamaparvovirus*, *Brevihamaparvovirus*, *Hepanhamaparvovirus*, *Ichthamaparvovirus*, and *Chaphamaparvovirus* (6–8).

Owing to advancements in detection and sequencing technologies in recent years, chaphamaparvoviruses has been identified in numerous hosts, including bat (*Eidolon helvum*) parvovirus 2 (EHPV2) (9), Cachavirus (CachaV) (10), *Chaphamaparvovirus carnivoran2* (feline ChPV, termed as fechavirus) (11), chicken parvovirus 1 and 2 (12), porcine parvovirus 7 (13), murine chapparvovirus (14), simian parvovirus (15), murine kidney parvovirus (16), and Hedgehog chapparvovirus (17). Until recently, CachaV was considered to be originally reported in American dog feces in 2019, which raised concerns among researchers about the virus (10). Similar to other parvoviruses, the CachaV genome mainly consists of two open reading frames that encode a capsid protein (VP1) and a nonstructural replication protein (NS1) (18). In the United States, CachaV was detected in 80 of 2,053 fecal samples (3 of 203 stool samples from healthy dogs and 77 of 1767 stool samples from dogs with diarrhea), with an infection rate of 4.35%, marking the first report of the novel parvovirus (10). Subsequently, researchers in China identified two samples that were positive for CachaV from 171 cats with diarrhea, whereas the 378 samples from healthy cats were all negative (19). In a study conducted in Thailand, among five dead dogs, three tested positive for CachaV in the lung tissue sample and one in the intestinal tissue sample (20). These similar reports indicate that CachaV is mainly related to the intestinal tract or diarrhea syndrome. To understand the recent prevalence and evolution status of CachaV, we investigated dogs from central, eastern, and northern China; sequenced and analyzed their genome sequences to facilitate epidemiological studies and mutation analysis; and then assessed their prevalence with possible clinical significance.

## 2 Materials and methods

### 2.1 Sample preparation

Anal swabs (cotton swabs soaked in physiological saline and inserted 2–3-cm deep into the anus of the sampling dog; these swabs were gently rotated, applied inside the anus, and then inserted into a clean test tube) from 276 dogs (182 with diarrhea and 94 healthy) were obtained from pet hospitals in five provinces (Henan, Inner Mongolia, Jiangsu, Hubei, and Anhui) of China between 2020 and 2023.

### 2.2 Viral DNA/RNA extraction

Each swab was washed with 1 mL of phosphate-buffered saline solution (0.01 mol/L), and 200 μL of suspensions were separated for viral DNA/RNA extraction using Simply Viral DNA/RNA Coextraction Kit (Bioer Biotechnology, Inc., Hangzhou, China) according to the manufacturer’s instructions. The extracted DNA and RNA were stored at −80°C for future use.

### 2.3 Pathogen screening

As previously reported, CachaV detection was performed using nested polymerase chain reaction (nt-PCR) as follows: the first PCR used the outer primer set IF (5′- CAACTAGCCGAATGCAGGGA-3′) and IR (5′-CGATAACATCCCCGGACTGG-3′) and the nested PCR used the inner primer set IF (5′-AGCTCAGTTTGGCCCAGATC-3′) and IR (5′-AGAGGGATCGCTGGATCTGT-3′) (10). In addition, canine parvovirus (CPV-2) (21), canine distemper virus (CDV) (22), canine coronavirus (CCoV) (23), and canine bufavirus (CBuV) (24) were screened in these collected samples using PCR/RT–PCR as previously described. The infection and co-infection statuses of the samples were summarized and displayed using UpSet plot packages (25). The prevalence of CachaV was compared between healthy and diarrheal dogs using Fisher’s exact test via GraphPad Prism 9.5 (San Diego, California, United States). Statistics were deemed significant at *p* < 0.05.

### 2.4 Genome sequencing of CachaV

The nearly complete genome sequences of the nine CachaVs were amplified using specific primers as described previously (19). Overlapped amplification of CachaV genome segments was performed using PCR in a 20-μL reaction system containing a template (>100 ng/L), 10 pmol forward/reverse primer sets, and Ex-Taq polymerase (TaKaRa Biotechnology Co., Ltd., Dalian, China). The reaction procedure was as follows: predenaturation at 94°C for 5 min; followed by 35 cycles of combined denaturation at 94°C for 45 s, annealing at 56°C for 45 s, extension of 72°C for 70 s; and final extension at 72°C for 10 min. After ligation of the PCR amplicons using ClonExpress Ultra One Step Cloning Kit (Vazyme Biotechnology Co. Ltd., Nanjing China), positive clones were sent for sequencing to Generalbiol -Biotechnology, Chuzhou, China.

### 2.5 Identification, recombination, and phylogenetic analysis

To analyze the genome sequence of CachaV and demonstrate its phylogenetic relationship, the sequence segments were assembled using DNAStar7.0 software (DNASTAR Inc., Madison, WI, United States) and aligned with all chaphamaparvoviruses. We used BioAider kit for the differential analysis of the 9 studied CachaV strains and 24 CachaV strains. A phylogenetic evolutionary tree was constructed using the genomes of the 9 studied CachaV strains; 24 CachaV strains detected in dogs, wolves, or cats in China or other countries (Supplementary Table S1). According to the MODELS program in MEGA 11 software, the HKY + G + I model, JTT + G model, and JTT + G model were used for the whole genome phylogenetic tree, NS1 phylogenetic tree, and VP1 phylogenetic tree, respectively. Evolutionary trees were further constructed based on the amino acid (aa) sequences of VP1 and NS1 using 1,000 guided replicates and the maximum-likelihood method via MEGA 11 software (26). Furthermore, recombination was predicted in the strains evaluated in this study using RDP4. Potential recombination events detected via three or more programs along with the identity analysis of the parents were considered potential events, with the highest acceptable *p*-value cutoff of 0.05 (27). RDP4 is a computer program typically used for recombination prediction; it includes guided scan, MAXCHI, mosaic, 3SEQ, gene cloning, LARD, and SISCAN in addition to the traditional RDP method that can predict recombination (28).

### 2.6 Protein mutation, antigen epitopes, and tertiary structure prediction

Based on the Meg-Align results, the aa sequences of NS1 and VP1 of the nine studied strains were compared with the reference CachaV strains identified in dogs or cats. Antigenic epitope prediction was performed using DNAMAN 5.2.2 for the resulting and reference strains (LynnonBiosoft, America) (Supplementary Table S2). To understand the molecular characteristics of the coding proteins, IDEXX1 (accession number: MH893826) and two variant CachaV strains (CHN230216 and CHN220916) were subjected to structural homology modeling for NS1 and VP1 using SWISS-MODEL2 and were visualized using PyMOL Molecular Graphics System 2.3 (DeLano Scientific LLC, America).

## 3 Results

### 3.1 Positive rate and co-infection with CachaV

**Figure 1:**
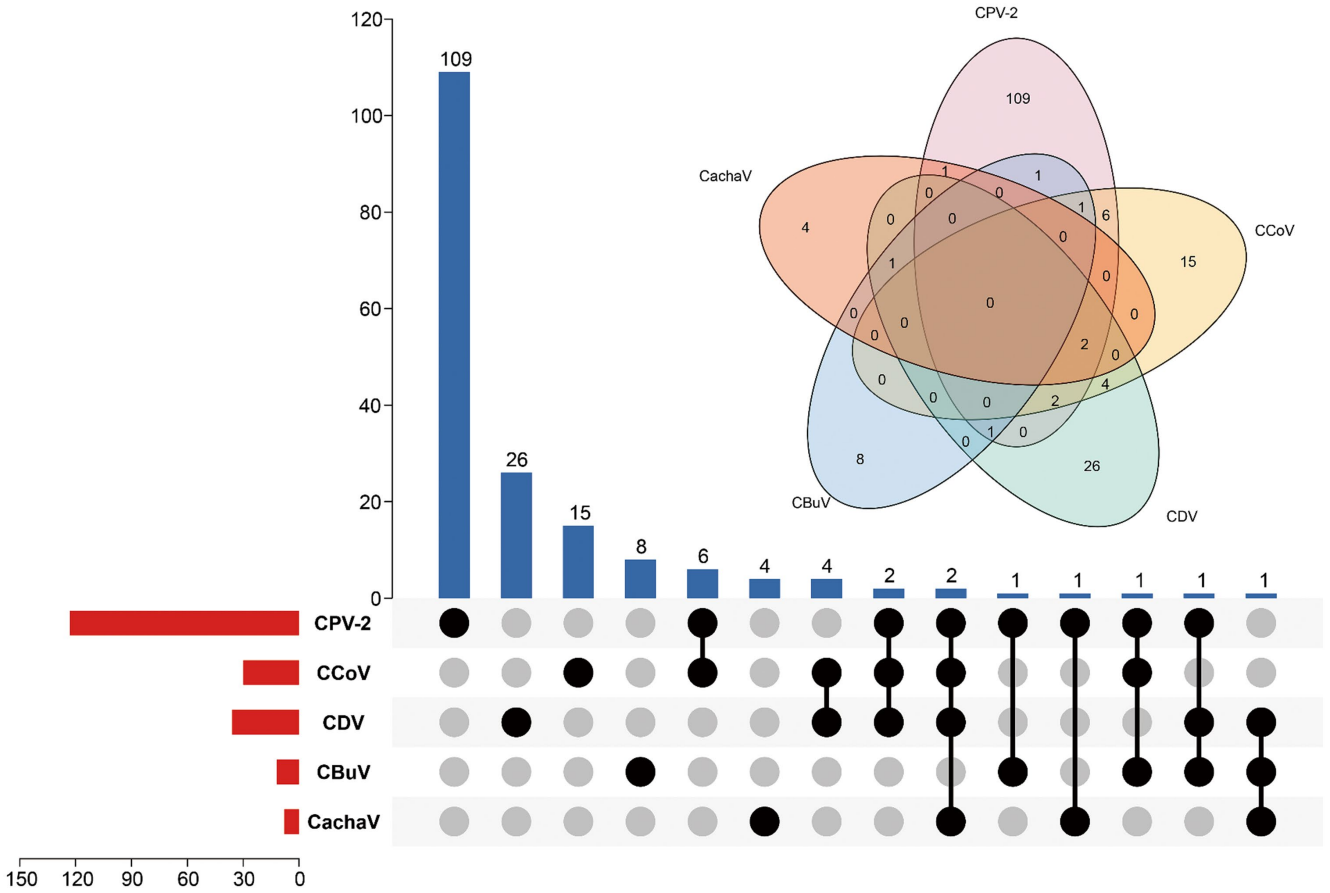
Infection status for CachaV, CPV-2, CCoV, CDV, and CBuV in samples from dogs with diarrhea. The upper bars indicate the numbers of positive samples in each group. The lower bars indicate the numbers of positive samples for each virus. The dotted line on the lower right indicates the types of infections.

Among the 276 samples, 9 (3.3%) tested positive for CachaV after viral screening, including 8 (8/182, 4.4%) from dogs with diarrhea in Henan and Jiangsu provinces and 1 (1/94, 1.1%) from a healthy dog in Anhui province. Notably, more than half of the total positive cases were detected in Henan and Jiangsu provinces. Table 1 shows the clinical information of the CachaV-positive dogs. Statistical analysis revealed no association between CachaV infection and clinical symptoms (*p* > 0.05). Meanwhile, two dogs with diarrhea were quadruply infected (CachaV + CPV-2 + CCoV + CDV), one dog with diarrhea was triply infected (CachaV + CDV + CBuV), and one dog with diarrhea was dually infected (CachaV + CPV-2). The infection and co-infection statuses of each pathogen screened are shown via UpSet and Venn plots in Figure 1.

**Table 1:** Details of the Cachavirus strains identified in this study.

Strain	Accession nos.	Health status	Age (months)	Province	Year
CHN210713	PP179517	Diarrhea	8	Henan	2021
CHN211026	PP179518	Diarrhea	9	Henan	2021
CHN220318	PP179519	Diarrhea	10	Henan	2022
CHN220916	PP179520	Diarrhea	10	Jiangsu	2022
CHN221119	PP179521	Diarrhea	8	Henan	2022
CHN230216	PP179522	Diarrhea	8	Henan	2023
CHN230409	PP179523	Diarrhea	8	Jiangsu	2023
CHN230521	PP179524	Diarrhea	10	Jiangsu	2023
CHN230827	PP179525	Healthy	11	Anhui	2023

### 3.2 Sequence identity analysis

The nine identified strains had the same genome structure as IDEXX1, each with two main codes: NS1 (663 aa) and VP1 (504 aa). Notably, the aa sequence identities of NS1 and VP1 in the nine CachaV strains with IDEXX1 were 97.89–98.94% and 97.42–99.8%, respectively. Compared with the 24 previously reported CachaV strains, the aa sequence identities of NS1 and VP1 in the 9 obtained CachaV strains were 97.59–99.55% and 96.23–99.8%, respectively. Moreover, the NS1 of CHN230521 showed the highest identity (99.85%) with NWT-W88 and CP-T015, whereas CHN220318 showed the lowest identity (96.23%) with OM640109. For the VP1 protein, CHN230521 exhibited the highest identity (100%) with NWT-W88 (accession no.: OK546101, originated from wolf, Canada, 2009), CP-R107C (accession no.: OP225937), CP-T015 (accession no.: OP225942), and CP-T046 (accession no.: OP225944), whereas CHN220916 showed the lowest identity (95.83%) with CY56 (accession no.: OM640109).

### 3.3 Phylogenetic analysis

**Figure 2:**
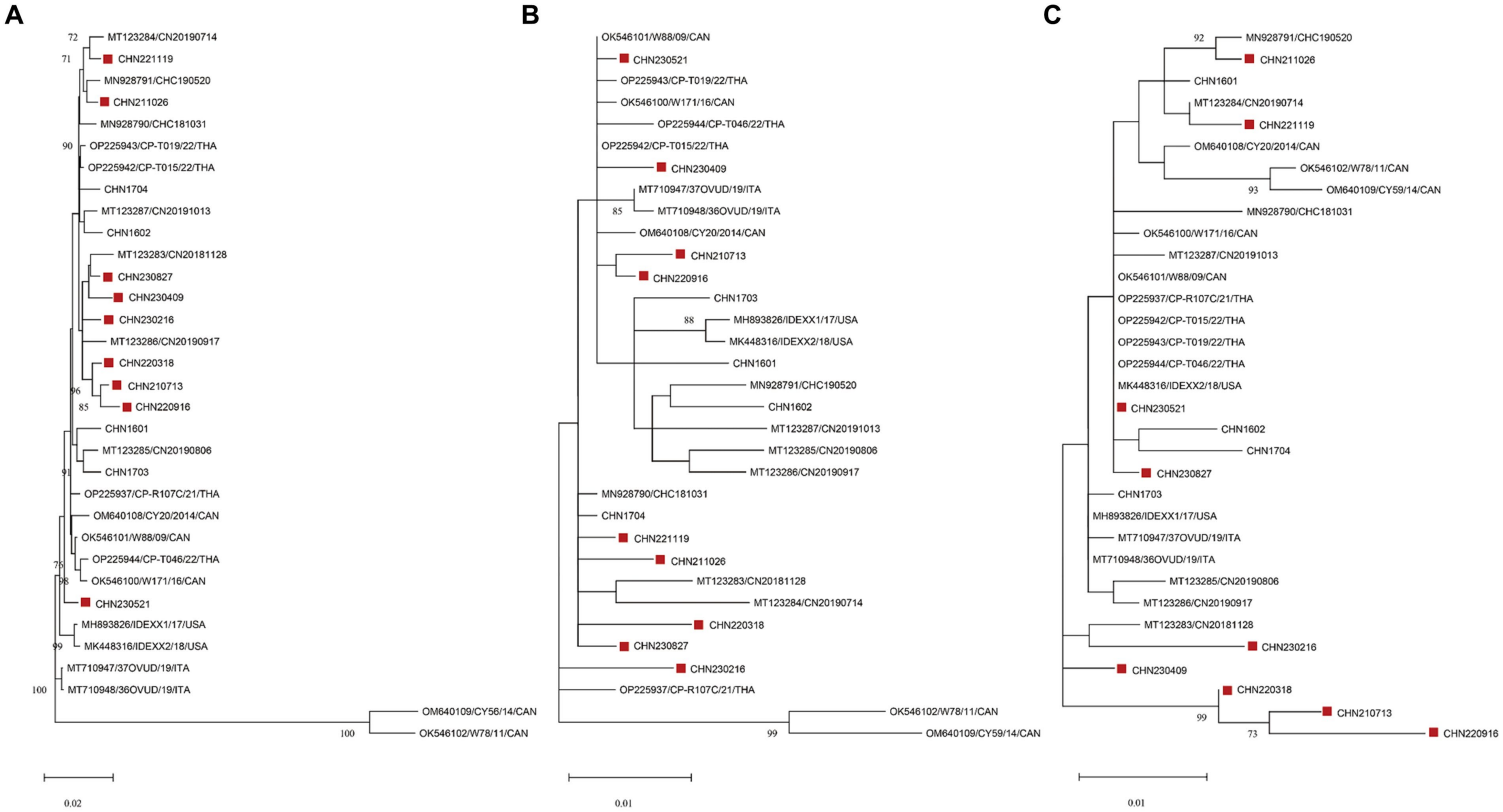
Maximum-likelihood trees based on genome sequences **(A)** deduced from NS1 **(B)** and VP1 **(C)** amino acid sequences of CachaVs. The CachaV strains obtained in this study are marked with red rectangles.

Phylogenetic analysis was performed using the genome sequences and aa sequences of NS1 and VP1 in the 9 identified strains and 24 CachaV strains (Figure 2). As shown in the evolution trees, CHN230521were closely related to some CachaV strains collected in Thailand, Canada and the United States, and the remain eight obtained strains mainly clustered with Chinese CachaV strains and displayed different branching, respectively. Most strains obtained in this study belonged to the same branch with the two cat-originated strains previously reported in China. Notably, no obvious recombination signal was observed in our comparative analysis with all chaphamaparvoviruses, despite the complex whole genome evolution of these nine strains.

### 3.4 Mutation of NS1 and VP1 proteins

**Figure 3:**
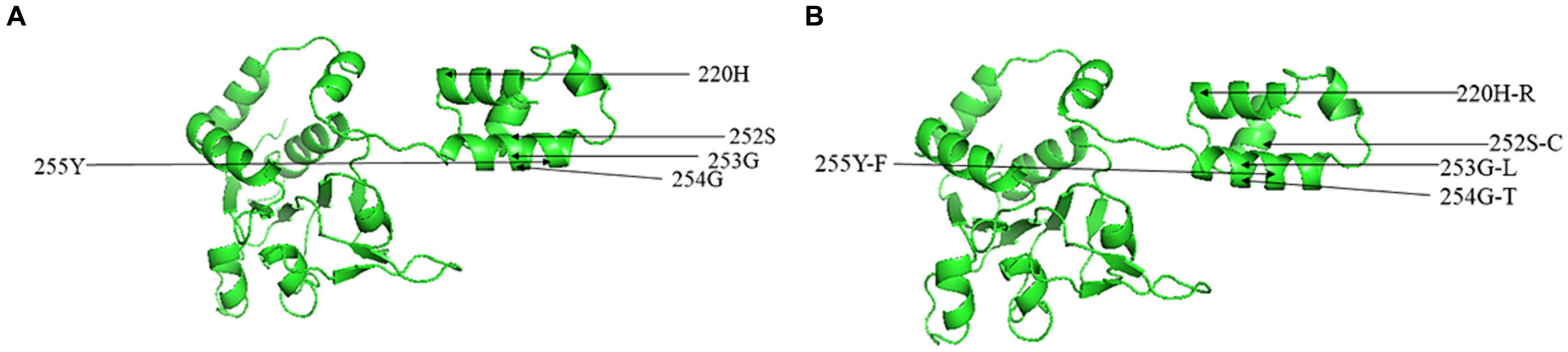
Predicted three-dimensional model of NS1 for CachaV strain of IDEXX1 **(A)** and CHN230216 **(B)**.

Through alignment of the derived aa sequences of the NS1 protein between the obtained CachaV and 24 CachaV strains, the following mutation sites were detected only in the nine obtained strains: Met104Leu, Asn207Asp., His220Arg, Gln469Arg, and Gly519Ser (Supplementary Table S3). The CHN210713 and CHN220916 strains showed unique site mutations at 99 (Phe → Ser), whereas the remaining seven strains had undergone aa changes at 456 (Leu → Thr). By comparing the aa sequences of CachaV-NS1, continuous mutations of SerGlyGlyTyr252-255CysLeuThrPhe were identified, which were common in other CachaV reference strains detected in China (Supplementary Table S4). For NS1, the CHN210713 strain identified in this study had mutations at loci 131 (Phe → Ser), 238 (Asp→Gly), and 247 (Val → Ala). Notably, the mutations generated at N9D in VP1 were present only in the CHN220916 and CHN230216 strains. To better understanding these mutation sites, mutations related to the tertiary structure change in NS1 were predicted and are displayed in Figure 3. The NS1 tertiary structure is predicted to vary at S252C which caused the change ofα-helix and random coil region, whereas these sites in VP1 have not been predicted to undergo structural changes.

## 4 Discussion

The first report related to CachaV detected in healthy dogs from the United States in 2017 revealed that dogs can serve as reservoir hosts for this virus without clinical symptoms (10). Currently, CachaV infection is mainly related to clinical symptoms of diarrhea, and the virus has been mainly reported in the United States, Italy, Canada, China, and Thailand (10, 20, 21, 29, 30). According to a report related to CachaV in northeast China, the positive rate of CachaV in samples from healthy dogs and dogs with diarrhea was 10% (4/40) and 6.3% (18/285) (31). In this study, nine samples tested positive for CachaV; the positive rate of CachaV in healthy dogs was (1/94, 1.1%), whereas the positive rate of CachaV in dogs with diarrhea was (8/182, 4.4%). Our statistical analysis showed no correlation (*p* > 0.05) between the presence of viruses and diarrhea and no statistical difference. In two studies in Canada, (i) 8 of 303 (2.6%) spleen samples from wolves tested positive for CachaV (30) and (ii) 3 of 87 (3.5%) spleen samples from coyotes tested positive for CachaV (32). In the past few years, we have tested positive samples from nine dogs and two cats, all of which showed symptoms of diarrhea (19, 33, 34). The global spread of CachaV has increased concerns worldwide. However, the evolution characteristics of CachaV remain relatively limited.

To further explore the evolutionary trend of CachaV strains, we performed phylogenetic analyses. In this study, according to the evolution trees, eight of nine identified strains belonged to the same clade as the dog–cat CachaV already reported in China, which was also observed in wolves, indicating the genetic relationship and distance between ChPV strains detected in other organisms (7). Based on the evolution tree constructed using genome sequences, the CHN230521 strain was closely related to the strain isolated from dogs in Canada (NWT-W171 and NWT-W88) and the United States (IDEXX2); however, the reasons for the similarity in these strains are not clear and need to be verified in future studies. Furthermore, no obvious recombination signal was observed in our comparative analysis with the reference strains, suggesting that site mutations remain the main evolutionary driver.

The NS1 protein in the nine studied strains exhibited unique mutations at the G253L locus compared with the IDEXX1 strain and Chinese dog strains. Compared with the antigenic site predictions of VP1 protein, we identified several site mutations of antigenic significance that may provide evidence for vaccine-strain selection in the future. Similar to the finding of a previous study in China, NS1 mutations occurred at both G254T and Y255F loci of Chinese strains, indicating that the determining region might have a common origin. For VP1, the CHN230513 strain differed from the IDEXX1 strain only at V265L, whereas CHN220916 varied significantly from the IDEXX1 strain and harbored the highest number of mutation sites and consecutive mutations at D278G–S279G. Notably, the site mutations at S77F, T152A, and D238G were only detected in the dog-originated strains. We found that the tertiary structure models varied in strains with more mutated sites. However, these are speculations, and further studies are warranted to demonstrate whether these mutations change the protein function. In addition, the reasons for these occurrences remain unknown. Mutational analysis revealed no significant differences between the VP1 model structure of the CHN220916 strain and IDEXX1 strain. A broader and more systematic study on the patterns and specific structural characteristics of CachaV mutations is warranted.

## 5 Conclusion

In conclusion, one and eight CachaV strains were identified in healthy and diarrheic dogs, respectively. No recombination events were predicted, and special mutation sites were detected in both VP1 and NS1 proteins. This study may contribute to the research on the monitoring and evolution of CachaV worldwide.

## Data availability statement

The datasets presented in this study can be found in online repositories. The names of the repository/repositories and accession number(s) can be found in the article/Supplementary material.

## Ethics statement

The animal studies were approved by sample collection was approved by the pet owner and Nanyang Normal University Animal Care Committee (No. 14027). The studies were conducted in accordance with the local legislation and institutional requirements. Written informed consent was obtained from the owners for the participation of their animals in this study.

## Author contributions

CL: Data curation, Investigation, Writing – review & editing. XT: Investigation, Software, Writing – original draft. HZ: Investigation, Methodology, Writing – review & editing. JJ: Conceptualization, Supervision, Writing – review & editing. LY: Funding acquisition, Supervision, Writing – review & editing.
