# Supplementary material for: Molecular epidemiological investigation and recombination analysis of Cachavirus prevalent in China
Source: Front Vet Sci. 2024 May 1;11:1375948. doi: 10.3389/fvets.2024.1375948 (PMC11094709; doi:10.3389/fvets.2024.1375948)
Supplement: Supplementary file 1 [file Table_1.docx]

Supplementary Material

**Molecular epidemiological investigation and recombination analysis of** **Cachavirus prevalent in China**

Chaoliang Leng^1^, Xiang Tian^1^, Hongyue Zhai^1^, Jun Ji^1,^*, Lunguang Yao^1^

**Correspondence:** Corresponding Author: [jijun020@126.com](mailto:jijun020@126.com)

**Supplementary Table 1.** Information about sequences of reference strains used in this study

| Strains | Host | Year | Country | Accession Nos. |
| --- | --- | --- | --- | --- |
| CN20181128 | Canine | 2018 | China | MT123283 |
| CN20190714 | Canine | 2019 | China | MT123284 |
| CN20190806 | Canine | 2019 | China | MT123285 |
| CN20190917 | Canine | 2019 | China | MT123286 |
| CN20191013 | Canine | 2019 | China | MT123287 |
| CHN1601 | Canine | 2016 | China | UYR58345 |
| CHN1602 | Canine | 2016 | China | UYR58346 |
| CHN1703 | Canine | 2017 | China | UYR58349 |
| CHN1704 | Canine | 2017 | China | UYR58350 |
| IDEXX1 | Canine | 2017 | USA | MH893826 |
| IDEXX2 | Canine | 2018 | USA | MK448316 |
| 37OVUD | Canine | 2019 | Italy | MT710947 |
| 36OVUD | Canine | 2019 | Italy | MT710948 |
| CP-R107C | Canine | 2021 | Thailand | OP225937 |
| CP-T015 | Canine | 2022 | Thailand | OP225942 |
| CP-T019 | Canine | 2022 | Thailand | OP225943 |
| CP-T046 | Canine | 2022 | Thailand | OP225944 |
| CY56 | Canis latrans | 2014 | Canada | OM640109 |
| CY20 | Canis latrans | 2014 | Canada | OM640108 |
| W171 | Canis lupus | 2016 | Canada | OK546100 |
| W88 | Canis lupus | 2009 | Canada | OK546101 |
| W78 | Canis lupus | 2011 | Canada | OK546102 |
| CHC181031 | Feline | 2018 | China | MN928790 |
| CHC190520 | Feline | 2019 | China | MN928791 |

**Supplementary Table 2.** Predicted antigen epitopes of VP1 protein of the Cachavirus strains identified in this study

| Amino acid sites | | Amino acid sequence | Score |
| --- | --- | --- | --- |
| 19–25 | PYVYPNN | | 1.062 |
| 28–34 | ATIVAAE | | 1.127 |
| 54–59 | HFIT | | 1.038 |
| 69–83 | EAYHVKGYSVIVYNP | | 1.126 |
| 86–93 | MTQQLAIQ | | 1.060 |
| 151–159 | RTILPIYSW | | 1.100 |
| 169–177 | DHTFSVDLT | | 1.088 |
| 179–186 | SGSAVWPA | | 1.078 |
| 196–202 | PTGCFWD | | 1.064 |
| 242–255 | KWAPYVHDNPFLKY | | 1.086 |
| 262–270 | GSYVVAPQD | | 1.155 |
| 288–307 | DKKCDYTIPDLSFLPIVPMS | | 1.172 |
| 316–321 | STAASD | | 1.079 |
| 337–353 | QYKYPPTQCFIKGLPLF | | 1.110 |
| 364–378 | TQGCFQVILHLACKK | | 1.196 |
| 382–387 | RYYAPT | | 1.035 |
| 455–466 | ITAPIKELVNLI | | 1.099 |
| 491–496 | IEVITA | | 1.054 |

**Supplementary Table 3.** Representative mutations in NS1 protein of Chinese Cachavirus (underlined strain name stands for strains evaluated in this study) compared with those of the prototype strain of IDEXX1 and Cachavirus

| Strains |  | Substitution of amino acid residues | | | | | | | | | | | | |
| --- | --- | --- | --- | --- | --- | --- | --- | --- | --- | --- | --- | --- | --- | --- |
|  |  | 58 | 99 | 220 | 252 | 253 | 254 | 255 | 456 | 457 | 469 | 603 | 605 | 607 |
| IDEXX1 | | S | F | H | S | G | G | Y | L | D | Q | G | A | R |
| CHN210713 | | - | S | - | C | L | T | F | - | N | - | R | - | G |
| CHN211026 | | A | - | - | C | L | T | F | - | N | - | R | - | G |
| CHN220318 | | - | - | - | C | L | T | F | T | N | - | R | - | G |
| CHN220916 | | - | S | - | C | L | T | F | T | - | - | R | - | G |
| CHN221119 | | - | - | - | C | L | T | F | T | N | - | R | - | G |
| CHN230216 | | - | - | R | C | L | T | F | T | N | R | R | T | G |
| CHN230409 | | - | - | - | C | L | T | F | T | - | - | R | V | G |
| CHN230521 | | - | - | - | C | L | T | F | T | - | - | R | - | G |
| CHN230827 | | - | - | - | C | L | T | F | T | N | - | R | - | G |
| CHC181031 | | - | - | - | C | L | T | F | - | N | - | R | - | G |
| CHC190520 | | - | - | - | - | V | T | F | - | N | - | R | - | G |
| CN20191013 | | - | - | - | - | V | T | F | - | N | - | R | - | G |
| CN20190917 | | - | - | - | - | V | T | F | - | N | - | R | - | G |
| CN20190806 | | - | - | - | - | V | T | F | - | - | - | R | - | G |
| CN20190714 | | - | - | - | C | L | T | F | - | N | - | R | - | G |
| CN20181128 | | A | - | - | C | L | T | F | - | N | - | R | - | G |
| CHN1601 | | - | - | - | - | V | T | F | - | - | - | R | - | G |
| CHN1602 | | - | - | - | - | V | T | F | - | N | - | R | - | G |
| CHN1703 | | - | - | - | - | V | T | F | - | - | - | R | - | G |
| CHN1704 | | - | - | - | C | L | T | F | - | N | - | R | - | G |
| CP-R107C | | - | - | - | C | L | T | F | - | - | - | R | - | G |
| CP-T015 | | - | - | - | C | L | T | F | - | - | - | R | - | G |
| CP-T019 | | - | - | - | C | L | T | F | - | - | - | R | - | G |
| CP-T046 | | - | - | - | C | L | T | F | - | - | - | R | - | - |

**Supplementary Table 4.** Representative mutations in VP1 protein of Chinese Cachavirus (underlined strain name stands for strains evaluated in this study) compared with those of the prototype strain of IDEXX1

| Strains |  | Substitution of amino acid residues | | | | | | | | | | | | | | | | | | | | | | |
| --- | --- | --- | --- | --- | --- | --- | --- | --- | --- | --- | --- | --- | --- | --- | --- | --- | --- | --- | --- | --- | --- | --- | --- | --- |
|  |  | 9 | 30 | 77 | 131 | 152 | 177 | 238 | 247 | 265 | 278 | 279 | 308 | 326 | 365 | 366 | 418 | 431 | 449 | 470 | 484 | 493 | 495 | 499 |
| IDEXX1 | | N | L | S | F | T | T | D | V | V | D | S | W | Y | Q | G | R | L | R | K | H | V | T | A |
| CHN210713 | | - | S | - | S | - | - | G | A | - | G | G | R | - | - | A | - | P | - | R | - | - | - | S |
| CHN211026 | | - | - | - | - | - | A | - | - | L | - | - | - | - | R | G | - | - | K | - | P | L | - | - |
| CHN220318 | | - | - | - | - | - | - | G | A | - | G | - | R | - | - | G | - | P | - | R | - | - | - | S |
| CHN220916 | | D | - | - | - | A | - | G | A | - | G | G | R | - | Q | A | - | - | - | R | - | - | - | S |
| CHN221119 | | - | - | - | - | - | - | - | - | L | - | - | - | - | - | G | - | - | K | - | - | L | S | - |
| CHN230216 | | D | - | F | - | - | - | - | - | - | - | - | - | N | - | G | G | - | - | - | - | - | - | - |
| CHN230409 | | - | - | - | - | - | - | - | - | - | - | - | - | - | - | G | G | - | - | R | - | - | - | - |
| CHN230521 | | - | - | - | - | - | - | - | - | L | - | - | - | - | - | G | - | - | - | - | - | - | - | - |
| CHN230827 | | - | - | - | - | - | - | - | - | L | - | - | - | N | - | G | - | - | - | - | - | - | - | - |
| CHC181031 | | - | - | - | - | - | - | - | - | L | - | - | - | - | R | G | - | - | K | - | P | L | - | - |
| CHC190520 | | - | - | - | S | - | - | - | - | L | - | - | - | - | - | G | - | - | K | - | - | - | - | - |
| CN20191013 | | - | - | - | - | - | - | - | - | L | - | - | - | - | - | G | - | - | - | - | - | - | - | - |
| CN20190917 | | - | - | - | - | - | - | - | - | - | - | - | - | - | - | G | - | - | - | - | - | - | - | - |
| CN20190806 | | - | - | - | - | - | - | - | - | - | - | - | - | - | - | G | - | - | - | - | - | - | - | - |
| CN20190714 | | - | - | - | - | - | - | - | - | L | - | - | - | - | - | G | - | - | K | - | - | L | - | - |
| CN20181128 | | - | - | - | - | - | - | - | - | - | - | - | - | N | - | G | - | - | - | R | - | - | - | - |
| CHN1601 | | - | - | - | - | - | - | - | - | L | - | - | - | - | - | G | - | - | K | - | - | L | - | - |
| CHN1602 | | - | - | - | - | - | - | - | - | L | - | - | - | - | - | G | - | - | - | - | - | - | - | - |
| CHN1703 | | - | - | - | - | - | - | - | - | - | - | - | - | - | - | G | - | - | - | - | - | - | - | - |
| CHN1704 | | - | - | - | - | - | - | - | - | L | - | - | - | - | - | G | - | - | - | R | - | - | - | - |
| CP-R107C | | - | - | - | - | - | - | - | - | - | - | - | - | - | - | - | - | - | - | - | - | - | - | - |
| CP-T015 | | - | - | - | - | - | - | - | - | - | - | - | - | - | - | - | - | - | - | - | - | - | - | - |
| CP-T019 | | - | - | - | - | - | - | - | - | - | - | - | - | - | - | - | - | - | - | - | - | - | - | - |
| CP-T046 | | - | - | - | - | - | - | - | - | - | - | - | - | - | - | - | - | - | - | - | - | - | - | - |
